# Cognitive functioning as an underutilized determinant of treatment planning: a review in alcohol use disorder

**DOI:** 10.3389/fpsyg.2026.1913195

**Published:** 2026-09-08

**Authors:** Emmanuel Streel, Madison Jones, M. Florencia Iulita

**Affiliations:** Department of Medical Affairs, Altoida Inc., Washington, DC, United States

**Keywords:** alcohol, alcohol use disorder (AUD), cognition, cognitive domains, neuropsychology

## Abstract

**Objective:**

Cognitive functioning is a critical determinant of treatment engagement, learning, and functional recovery across psychiatric disorders, yet it remains insufficiently integrated into treatment planning. While cognitive impairment is well documented in alcohol use disorder (AUD), its role is most often limited to a prognostic marker rather than an actionable input into clinical decision-making. This review examines how cognitive and neuropsychological functioning have been assessed and used to inform treatment planning in AUD.

**Methods:**

A PubMed (MEDLINE) search was performed on primary studies involving adults with AUD leading up to 2026, in which cognitive or neuropsychological assessment influenced, or was intended to influence, treatment planning or clinical decision-making. Studies assessing cognition solely as an outcome were excluded. Twenty-three (23) studies met inclusion criteria. Study designs included observational cohorts, feasibility trials, and controlled trials. Cognitive domains, assessment approaches, and treatment-related outcomes were synthesized narratively.

**Results:**

Across studies, cognitive functioning, particularly amongst executive function, attention, and memory domains, were consistently associated with treatment engagement, retention, and functional outcomes. Still, cognition was most often examined as a prognostic factor as opposed to an explicit input to treatment allocation or adaptation. Only a few studies incorporated cognitive assessment into treatment orientation or treatment modality selection. Assessment methods varied widely, limiting comparability and scalability.

**Conclusions:**

Cognitive functioning is clinically relevant, but current evidence characterizes it predominantly as a prognostic marker rather than a demonstrated, actionable input into treatment planning. Dedicated research testing cognition-informed treatment allocation and adaptation is needed before its routine incorporation into AUD care can be recommended.

## Introduction

Alcohol use disorder (AUD) is a leading cause of preventable morbidity and mortality worldwide and is associated with substantial neuropsychological sequelae. Chronic use of alcohol has been consistently linked to structural and functional brain changes (Yang et al., 2022; Oscar-Berman and Marinkovic, 2003; Sullivan and Pfefferbaum, 2005) with long term, excessive use frequently correlated with brain damage and neurocognitive functioning anomalies. The degree of impairment typically observed can be either mild or severe, persisting beyond brief periods of intoxication and involving specific difficulties in learning, memory, visuospatial abilities, executive function and social cognition (Heirene et al., 2018; Stavro et al., 2013; Le Berre et al., 2017).

The cognitive consequences of AUD are especially relevant in older adults, for whom age-related cognitive decline, reduced cognitive reserve, and multimorbidity may interact with alcohol-related neurotoxicity to exacerbate functional impairment (Sachdeva et al., 2016; Rao et al., 2022). As alcohol use continues and/or increase in these populations, treatment services have been increasingly confronted with patients whose cognitive capacity is insufficient in meeting the implicit demands of standard psychotherapeutic interventions. This is particularly important when considering the need for patients to relearn how to navigate a new environment in the absence of alcohol (or drugs), a process we have previously conceptualized as “*learning the language of abstinence*.” (Brewer and Streel, 2003). Nevertheless, cognitive function is rarely assessed or systematically incorporated into treatment planning in routine alcohol care (Verdejo-Garcia et al., 2019). Most evidence-based interventions for AUD, including cognitive–behavioral therapy, relapse prevention, and motivational approaches, implicitly assume intact capacities for attention, abstraction, learning, and/or inhibitory control. As such, treatment effectiveness is likely to be compromised, where these assumptions are not met, resulting in reduced engagement and premature dropout, even limiting potential benefit despite program adherence (Domínguez-Salas et al., 2016). While clinicians generally recognize the relevance of cognition in practice, our clinical experience suggests that cognitive findings are often insufficiently translated into individualized treatment planning, a pattern consistent with the limited evidence documenting how cognitive assessment informs management decisions in routine clinical care (Hallam et al., 2021).

Indeed, a growing body of research has observed associations between specific cognitive domains, however, there is considerable heterogeneity in cognitive impairment and its severity and studies have not identified a systematic pattern of cognitive deficits in persons with AUD (Stavro et al., 2013; Høiland et al., 2025; Bates et al., 2002). Cognition is predominantly examined as a prognostic factor, with few studies translating neuropsychological findings into concrete treatment adaptations or allocation strategies (Teichner et al., 2001; Rychtarik et al., 2017). Cognitive assessment practices also vary widely, ranging from brief screening measures to more comprehensive neuropsychological batteries, limiting comparability and clinical scalability. Recent advances in digital health and technology-enabled cognitive and functional assessment have raised interest in more scalable, repeatable, and ecologically valid approaches to measuring cognition (Lott et al., 2024) with translational opportunities in addiction settings. However, the extent to which these approaches address the practical barriers identified in current practice remains unclear. This review will examine the relevance of cognition in treatment planning, specifically: zeroing in on the cognitive domains most frequently implicated, the approaches used to assess cognitive functioning, the impact of cognition as a factor in mapping treatment processes, and the outcomes and limitations of these approaches.

In identifying consistent patterns and gaps in the evidence base, this review seeks to clarify why cognition remains historically underutilized in alcohol treatment planning and to inform future directions for more scalable and clinically actionable neurocognitive-oriented strategies.

## Methods

This review examined how cognitive or neuropsychological functioning is assessed and considered as a possible input to AUD treatment planning. The research question was: “*How is cognitive or neuropsychological status assessed and used to inform, guide, tailor, or determine AUD treatment planning?”* This review was conducted in accordance with PRISMA principles for study identification, screening, and selection, with a structured search strategy, predefined eligibility criteria, and transparent reporting of included studies. Included studies were deemed eligible if they included adults with AUD and described a treatment context in which cognitive or neuropsychological assessment influenced, informed, or was intended to inform clinical decision-making, including treatment selection, adaptation, intensity, setting, eligibility, readiness, or capacity to engage in treatment. Studies were not excluded on the basis of psychiatric comorbidity or comorbid substance use, provided AUD was the primary or predominant substance-related diagnosis under study; studies in which polysubstance use or another substance use disorder was the primary focus, with alcohol use addressed only incidentally, were excluded. It is important to note that studies in which cognition was assessed only as an outcome measure, without potential implications for treatment planning or delivery, were excluded. Studies focusing exclusively on neurobiology or imaging without treatment implications or animal studies were also excluded. Given the heterogeneity in study designs and outcomes, a formal quantitative risk-of-bias assessment was not performed; however, study characteristics and methodological limitations were qualitatively evaluated during the preparation of the result synthesis.

## Search strategy and information sources

A PubMed (MEDLINE) search was performed from all primary studies leading up to 2026, using keywords combining three concepts: cognitive status AND alcoholism AND treatment. For cognitive status, terms included “neuropsychological assessment” OR “neuropsychological evaluation” OR “cognitive screening” OR “neuropsychological tests” OR “cognition disorders” OR “cognition.” For AUD, terms included “alcohol use disorder” OR “alcohol dependence” OR “alcoholism” OR “Alcohol-Related Disorders.” For treatment planning/decision-making, terms included “treatment planning” OR “treatment selection” OR “clinical decision making” OR “treatment suitability” OR “ability to engage” OR “treatment outcome” OR “patient care planning” OR “rehabilitation.”

## Studies selection

**Figure 1:**
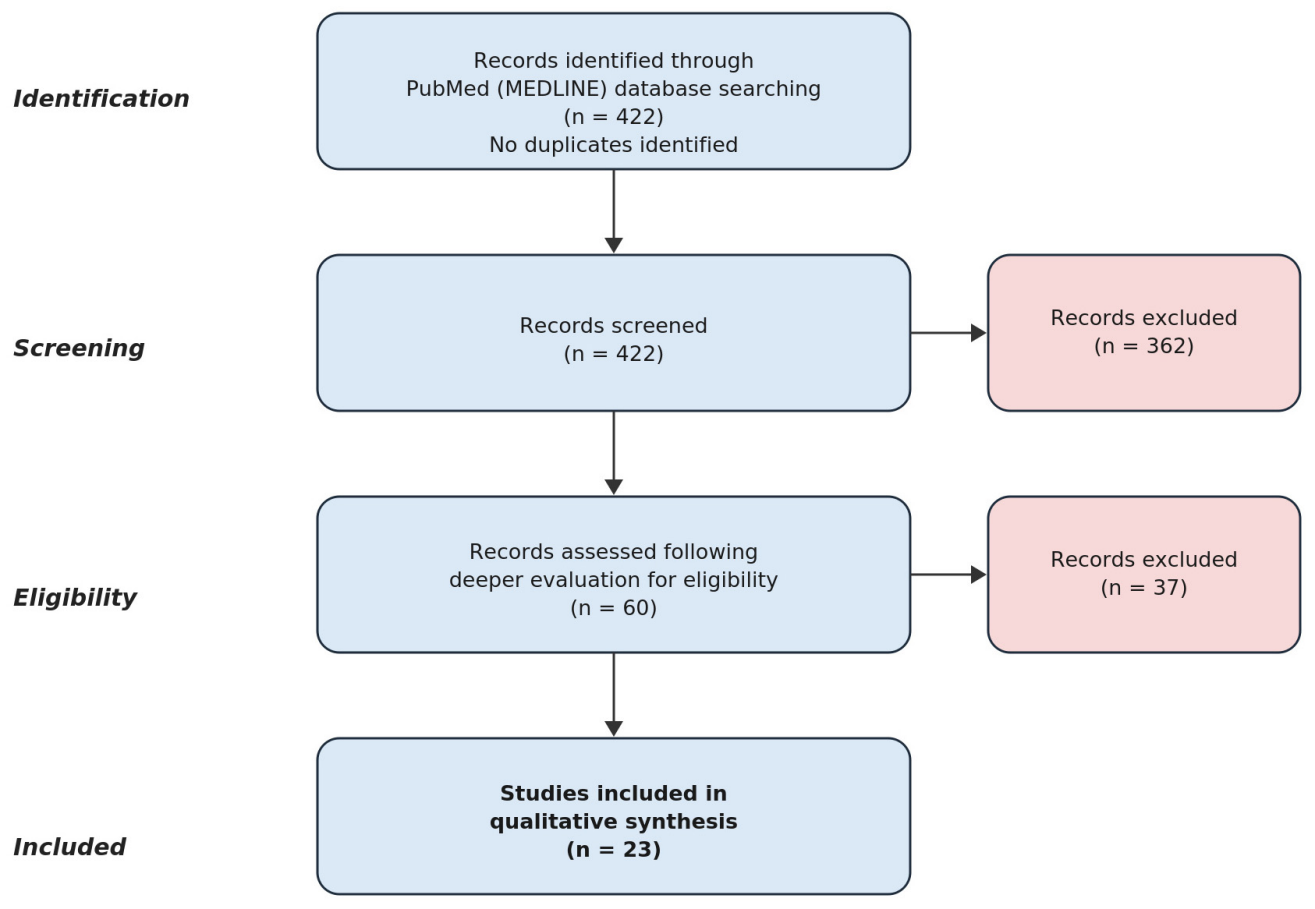
PRISMA flow diagram of study identification, screening, and inclusion.

Titles and abstracts of all retrieved records were screened for eligibility. Full texts were then reviewed to confirm that included studies explicitly addressed cognition as an input to treatment planning or clinical decision-making. The initial search identified 422 records (no duplicates). After title/abstract screening, 60 records were retained for inclusion; following deeper evaluation, 23 articles met full-text eligibility criteria, and were included in this paper and reviewed systematically (see Table 1). The study identification and selection process is summarized in Figure 1.

**Table 1:** List of studies selected.

References	Design	Cognitive domains assessed
Bates (1997)	Repeated-measures psychometric study	Executive function, memory, attention, visuospatial abilities
Teichner et al. (2001)	Observational	Global cognition, executive function
Moriyama et al. (2002)	Observational	Memory, attention, executive function
Lemke and Moos (2002)	Observational	Attention, memory, executive function
Block et al. (2003)	Prospective	Executive function, abstraction, memory
Fama et al. (2004)	Longitudinal	Memory, executive function
Verdejo-García and Pérez-García (2007)	Observational	Decision-making, executive control
Buckman et al. (2008)	Prospective	Executive function, memory
Le Berre et al. (2010)	Observational synthesis	Memory, executive function, visuospatial abilities
Sawayama et al. (2012)	Observational	Drinking-related cognitions (expectancies, self-efficacy, perceived control, perceived drinking problems).
Bell et al. (2016)	Controlled intervention	Verbal learning, memory
Schmidt et al. (2016)	Observational	Executive function
Rettie et al. (2018)	Qualitative	Metacognition
Loijen et al. (2018)	Observational	Executive function
Gunn et al. (2018)	Randomized controlled trial	Working memory/executive function
Rychtarik et al. (2017)	Randomized controlled trial	Cognitive control
Ihara et al. (2000)	Observational	Attention, memory
Block et al. (2003)	Prospective	Executive function
Schmid et al. (2021)	Observational	Executive function
Caballeria et al. (2022)	Pilot study	Attention, memory, executive function
Gamito et al. (2021)	Pilot RCT	Attention, cognitive flexibility, executive function
Rao et al. (2022)	Observational	Global cognition, executive function
Høiland et al. (2025)	Observational	Executive function

## Results

The final synthesis included 23 studies examining cognition in the context of AUD treatment. Study designs comprised cross-sectional neuropsychological profiling, prospective observational cohorts, allocation/feasibility studies, and pilot controlled trials. Most studies were conducted in inpatient or residential treatment settings, with fewer in outpatient care. Cognition was operationalized heterogeneously, ranging from brief screening measures to multi-domain neuropsychological batteries. Across the evidence base, cognition was examined primarily as a predictor or moderator of treatment engagement and outcomes, a target of intervention through cognitive remediation or training, and less frequently an explicit input into treatment planning or allocation. The clinical or treatment-related endpoints assessed were similarly heterogeneous. Most studies examined process-level endpoints—treatment retention or discontinuation, attainment of individualized treatment objectives, or functional/interpersonal outcomes, rather than treatment efficacy in a strict sense. A smaller subset examined drinking-related outcomes directly, including percentage of days abstinent, drinks per drinking day, or short-term abstinence following detoxification. Very few studies assessed relapse or long-term drinking outcomes at all.

## Relevance of cognition in alcohol treatment planning

Multiple studies have indicated that baseline cognitive functioning is closely associated with clinically relevant treatment outcomes. Early cohort work demonstrates that global cognitive functioning predicted treatment-related outcomes including discharge status and post-treatment adjustment, even after alcohol severity was accounted for (Lemke and Moos, 2002). Subsequent prospective studies reinforced the prognostic relevance of cognition, particularly executive functioning, for treatment persistence and functional recovery (Moriyama et al., 2002; Ihara et al., 2000). Executive dysfunction was further associated with poorer engagement or higher likelihood of treatment discontinuation. In a large prospective cohort, impairments in executive functioning predicted treatment dropout among individuals undergoing AUD treatment (Høiland et al., 2025). Similarly, attentional and working memory capacity have been observed to influence attainment of treatment objectives, suggesting that cognitive limitations may limit patients' abilities to benefit from cognitively demanding therapeutic components (Teichner et al., 2001). A small number of studies moved beyond prognostic association to build cognition directly into a treatment-planning framework, though with mixed evidence of benefit. Rychtarik et al. (2017) combined a brief processing-speed screening measure (Symbol Digit Modalities Test) with alcohol severity to classify clients a priori as needing inpatient vs. outpatient care, then randomized clients within this classification to test whether it predicted differential benefit by treatment setting; in this community-based replication, cognitive functioning did not significantly moderate drinking outcomes, and only alcohol severity was validated as a placement criterion. Teichner et al. (2001) described a program in which patients with impaired neuropsychological screening profiles were routinely assigned to a more basic version of several therapy groups, a genuine, low-burden translation of cognitive screening into programming decisions. However, the effectiveness of this accommodation was not directly tested, since group-level assignment was fully confounded with impairment status. The same study's strongest finding was prognostic rather than operational: baseline attention, not global cognitive impairment, was the most consistent predictor of the percentage of treatment objectives patients went on to attain.

## Cognitive domains considered relevant

Across the literature, executive functions (e.g., inhibition, cognitive flexibility, planning) emerged as the most consistently relevant cognitive domain. Executive impairment was identified as a predictor of real-world dysfunction (Verdejo-García and Pérez-García, 2007), including occupational outcomes (Moriyama et al., 2002), treatment discontinuation (Høiland et al., 2025) and differential benefit from treatment modalities (Block et al., 2003). Consistent with this heterogeneity, a cluster-analytic study identified three distinct executive-function profiles among recently detoxified patients with AUD, one largely unimpaired and two showing different patterns of deficit—suggesting that global group comparisons may obscure clinically meaningful subtypes (Schmid et al., 2021). Attention and processing speed were also linked to executive control particularly in regards to engagement and retention (Teichner et al., 2001; Rychtarik et al., 2017). Memory deficits, particularly verbal learning and episodic memory were commonly observed and associated with poorer treatment engagement and were identified as extremely relevant to treatment outcome and skill acquisition (Bell et al., 2016; Fama et al., 2004). That being said, several studies also suggested that memory impairment alone was insufficient in fully explaining treatment outcomes (Block et al., 2003; Buckman et al., 2008), highlighting the clinical complexity and consideration necessary in approaching cognition in AUD. A subset of studies focusing on implicit cognitive processes, including attentional bias and implicit learning proved relevant to this discussion. In one study, attentional bias measures predicted short-term drinking outcomes following detoxification (Rettie et al., 2018) while in another, a more preserved implicit learning capacity was also proposed as a compensatory mechanism in individuals with explicit memory deficits (Fama et al., 2004). These outcomes suggest that treatment efficacy may rely upon intervention mechanisms in alignment with the preservation of patients' cognitive systems (Loijen et al., 2018).

Less frequently studied, though clinically relevant, domains including social cognition and metacognition provide valuable insights as well. Schmidt et al. (2016) identified impairments in social problem-solving and pragmatic social understanding among inpatients with AUD, noting potential implications for interpersonal functioning during treatment. Le Berre et al. (2010) observed a disconnect between subjective confidence and objective memory performance, indicating specific (e.g., metacognitive) deficits could potentially engender an overestimation of treatment readiness or learning capacity. Notably, these domains are rarely assessed in routine care despite their potential toward risk-prevention and treatment engagement.

## Approaches used to evaluate cognition

Several studies relied on brief screening or single-domain measures, including processing speed or attention testing, to operationalize cognition in treatment contexts (Teichner et al., 2001; Rychtarik et al., 2017). While these basic approaches demonstrated feasibility and the potential for integration into clinical workflows, they provided limited domain specificity. A small number of studies extended the construct of cognition beyond standard neuropsychological testing to self-reported drinking-related cognitions, such as perceived control and abstinence self-efficacy, which also predicted abstinence outcomes, illustrating variability not just in method but in how cognition itself was operationalized (Sawayama et al., 2012). Other studies employed a more complex approach, such as multi-domain neuropsychological batteries to asses various cognitive domains (Ihara et al., 2000; Block et al., 2003; Buckman et al., 2008). Though these approaches certainly enabled richer cognitive profiling and mechanistic insight, in real world practice, their resource-intensive design lacks replicability or standard use in routine clinical deployment. Other forms of assessments, like the Behavioral Assessment of the Dysexecutive Syndrome, were used to capture real-world executive dysfunction more directly. More recently, technology-based approaches have also emerged. Caballeria et al. (2022) demonstrated that a virtual reality (VR)–based cognitive training protocol was usable and acceptable among AUD patients, including those with cognitive impairment. A pilot RCT by Gamito et al. (2021) also demonstrated that VR-based cognitive training protocol during residential treatment could produce additional improvements in attention and cognitive flexibility that could positively impact abstinence.

## Treatment impact of considering cognition

Direct evidence that cognition-informed approaches improve alcohol-related outcomes is limited. Block et al. (2003) demonstrated that premorbid cognitive functioning could moderate the effectiveness of different treatment modalities. Teichner et al. (2001) found that baseline attention and depressive symptoms, rather than global cognitive impairment, predicted the degree to which patients went on to attain individualized treatment objectives, though the accompanying practice of assigning cognitively impaired patients to less demanding programming was not itself tested against outcomes.

Several studies also indicated that cognition can modify in what way recovery might occur. For example, Buckman et al. (2008) note that executive impairment increased the reliance on social network support for successful outcomes, suggesting that cognitively impaired individuals may benefit from more structured environments. Intervention studies further demonstrated that cognition is modifiable during treatment, with cognitive training improving specific domains like attention, memory, and executive functioning (Bell et al., 2016; Gamito et al., 2021). A randomized controlled trial of working memory training found that individuals with AUD showed comparable training gains to controls, but significantly lower adherence to the training protocol itself (Gunn et al., 2018). Notably, most intervention studies did not directly assess relapse or long-term drinking outcomes.

## Discussion

This review supports two related but distinct conclusions, which we treat separately throughout. First, cognitive functioning is robustly and consistently underutilized as an input to AUD treatment planning: cognitive impairment is well established as a determinant of treatment engagement, retention, and outcome, yet is rarely assessed for this explicit purpose in routine care. Second, whether routine implementation of cognition-informed treatment planning would itself improve clinical outcomes remains largely untested. The evidence reviewed here supports the first conclusion far more strongly than the second, and should not be read as justifying routine implementation on its own.

Across several study designs, executive functioning and attentional capacity emerged as the most frequently implicated domains, influencing treatment retention, attainment of therapeutic objectives, and real-world functioning (Høiland et al., 2025; Verdejo-García and Pérez-García, 2007; Buckman et al., 2008). Memory, implicit cognitive processing, social cognition, and metacognitive abilities were also shown to be clinically relevant, though were less consistently assessed (Fama et al., 2004; Schmidt et al., 2016; Le Berre et al., 2010). Despite this evidence of significant effect in AUD, cognition was rarely fully operationalized as an input into treatment planning (Høiland et al., 2025; Lemke and Moos, 2002).

Only two studies explicitly built cognitive assessment into a treatment allocation or level-of-care framework (Teichner et al., 2001; Rychtarik et al., 2017) and neither offers clear evidence that doing so improved patient outcomes. This disconnect highlights a persistent gap between the recognized role of cognition in AUD and its practical integration into alcohol treatment services. More broadly, few studies linked cognitive assessment to long-term clinical or functional outcomes at all (Bell et al., 2016; Gamito et al., 2021), underscoring that the evidence base remains predominantly associative rather than outcome-oriented.

The reviewed studies also suggest that cognitive impairment not only predict poorer outcomes but meaningfully limits treatment benefit. Executive dysfunction can limit patient's capacity to engage with cognitively demanding interventions, while attentional deficits can impair the encoding and retrieval of therapeutic material (Domínguez-Salas et al., 2016; Bates et al., 2006). Metacognitive impairments, including inaccurate self-assessment of learning and competence, may compromise treatment effectiveness by producing a discrepancy between perceived and actual readiness for change (Le Berre et al., 2010). Several studies have also suggested that cognitive capacity influences how individuals achieve recovery. Executive impairment was observed to increase reliance on external social support for successful outcomes, suggesting that cognitively impaired individuals may benefit more from structured environments or externally supported treatment models (Buckman et al., 2008). These findings highlight the need to better align treatment demands with patients' neuropsychological capacities rather than assuming uniform cognitive readiness.

Although a lesser focus, several important findings in older age populations were observed. For example, older aged individuals with AUD were more likely to exhibit cumulative cognitive vulnerability due to age-related decline and comorbidities linked to reduced cognitive reserve (Sachdeva et al., 2016; Rao et al., 2022). This overlap is not merely additive: in older adults, alcohol-related cognitive impairment can closely resemble, or coexist with, neurodegenerative conditions such as Alzheimer's disease and other dementias, including the Wernicke–Korsakoff spectrum, complicating differential diagnosis in routine clinical settings (Sachdeva et al., 2016; Zahr, 2024). Unlike most neurodegenerative dementias, alcohol-related cognitive impairment can show partial or substantial improvement with sustained abstinence—a distinguishing clinical feature with direct implications for treatment planning and prognosis, though one that is difficult to establish without extended follow-up or a reliable drinking history (Sachdeva et al., 2016; Zahr, 2024). Without careful differential diagnosis, older patients with reversible alcohol-related impairment risk being miscategorized as having a fixed or progressive neurodegenerative process, or vice versa, potentially resulting in inappropriate treatment planning or missed opportunities for cognitive recovery. In inpatient settings, where treatment intensity and cognitive demands are high, failure to account for cognitive limitations may disproportionately affect older patients, contributing to disengagement or suboptimal outcomes. This dearth of age-specific studies underscores a critical gap in the literature. Nonetheless, whether the consistency of cognitive–treatment associations across mixed-age samples extends to older adults remains an open question rather than an established finding; we present this as a hypothesis warranting dedicated age-specific research, rather than a conclusion supported by the current evidence.

We also identified substantial heterogeneity in cognitive assessment methods, limiting comparability across studies (Block et al., 2003; Buckman et al., 2008). Although shorter-length screening tools are a more accessible and feasible approach to assessing cognition, they lack the necessary sensitivity for consistent relevance to treatment engagement. Comprehensive neuropsychological batteries provide the much needed granularity of a patient's cognitive architecture, but are resource and skill-intensive, making them poorly suited to routine or repeated use in clinical settings.

Further, subjective insights (e.g., self-reported measures like questionnaires), though factored into cognitive functioning in treatment protocols, are unreliable and therefore limited in their utility (Le Berre et al., 2010). Additionally, common concerns of the potential unreliability of cognitive assessments during detoxification were not supported by psychometric evidence, with neuropsychological performance shown to be stable within the first few weeks of treatment (Bates, 1997). This is encouraging for treatment planning.

The reviewed studies clearly demonstrate that cognition is clinically relevant to AUD treatment engagement and outcomes, and that existing neuropsychological assessment approaches carry practical limitations: brief screening tools sacrifice domain specificity, while comprehensive batteries are resource-intensive and poorly suited to repeated use in routine care (Verdejo-Garcia et al., 2019; Bates et al., 2013). However, the reviewed literature does not establish that these assessment limitations are the primary reason cognition has not been more widely incorporated into treatment planning; other factors—including the limited evidence of actionable benefit discussed above, competing clinical priorities, and structural or resourcing barriers in treatment settings—may also contribute, and this review was not designed to adjudicate between them. Emerging digital and technology-enabled cognitive assessment tools have been proposed as one possible way to address the scalability and repeatability limitations of traditional approaches, and early studies suggest feasibility and acceptability among cognitively impaired individuals (Caballeria et al., 2022; Gamito et al., 2021). We note this as a direction warranting future study rather than a conclusion supported by the current evidence: no study in this review evaluated whether digital cognitive assessment improves treatment planning or patient outcomes in AUD, and this should not be inferred from the present findings.

The limitations of this review include heterogeneity in study designs, populations, and cognitive measures. Multiple studies included mixed-age participants and direct evidence from primarily older-aged cohorts remains scarce. Additionally, few studies directly evaluated alcohol-related outcomes following cognition-informed treatment adaptation. The included studies also varied in the extent to which psychiatric and substance-use comorbidities were present or characterized in study samples [e.g., (Teichner et al., 2001) sample included patients with comorbid cocaine dependence], which may have influenced neuropsychological findings independent of AUD itself and limits the ability to attribute observed cognitive effects to alcohol use specifically. This review's scope was deliberately limited to AUD, given its distinct neurotoxic profile, epidemiology, and treatment landscape relative to other substance use disorders. Polysubstance use is nonetheless common in clinical AUD populations, as reflected in several included samples with comorbid substance use [e.g., (Gunn et al., 2018; Teichner et al., 2001)]; a dedicated review examining cognitive functioning across substance use disorders more broadly, accounting for the differing cognitive profiles associated with different substances, would be a valuable complement to the present focused analysis.

Future research should prioritize prospective studies that integrate cognitive assessment directly into treatment decision-making to examine their longitudinal cognitive trajectories, allowing for the evaluation of downstream clinical and functional outcomes. Adaptive study designs incorporating scalable digital cognitive assessment may be particularly suited to addressing these challenges in real-world inpatient settings, as they align with broader movements toward personalized precision approaches in psychiatry.
